# Unusual cause of hemoptysis: A case report

**DOI:** 10.1016/j.amsu.2020.07.063

**Published:** 2020-08-25

**Authors:** J. Fijolek, K. Oniszh

**Affiliations:** a The Third Department of Pneumonology, National Tuberculosis and Lung Diseases Research Institute, Plocka St. 26, 01-138, Warsaw, Poland; b Department of Radiology, National Tuberculosis and Lung Diseases Research Institute, Plocka St. 26, 01-138, Warsaw, Poland

**Keywords:** Hemoptysis, Scoliosis, Surgery, Complications, Case report

## Abstract

Hemoptysis is defined as bleeding from the lower respiratory tract. It can be life-threatening and requires urgent investigation and intervention. Common causes of hemoptysis include bronchiectasis, tuberculosis, aspergilomas and malignancy. We present an unusual case of hemoptysis in a young woman with a history of surgery for scoliosis 18 years earlier.

A 30-years-old woman was admitted to our institution for recurring hemoptysis since one year. She had a history of scoliosis and had undergone antero-lateral Th7 through Th12 spinal fusion surgery 18 years earlier. The hemoptysis was slight and resolved spontaneously or after empirical antibiotic therapy, and was attributed to bronchitis. Computed tomography revealed spinal rod penetration into the lung resulting in injury, while the caudal edge of the rod migrated into the liver and the joining screws had entered the mediastinum. Hemoptysis was due to penetration of the rod into the lung. The patient underwent extensive surgery, which was successful.

The case highlights the need for thorough evaluation of patients with hemoptysis. Every incident of hemoptysis, even if minor, should be promptly investigated, because it can be life-threatening.

## Highlights

• This case demonstrates a rare cause of hemoptysis due to penetration of the rod inserted during surgery for scoliosis into the lung 18 years after surgery; this condition was accompanied by migration the caudal edge of the rod into the liver and displacement of the joining screws into the mediastinum threatening to damage the aorta.
• It highlights the need promptly and thoroughly evaluate patients with even slight hemoptysis because it can be life-threatening.
• It indicates the need for periodic follow-up of patients after scoliosis surgery to prevent or minimize its complications.

## 1 Introduction

Hemoptysis is defined as bleeding from the lower respiratory tract. It can be life-threatening and requires urgent investigation and intervention. The diagnostic procedures should identify both, the source, and the underlying cause. Common causes include bronchiectasis, tuberculosis, and malignancy; however many other conditions such as cardiac, rheumatologic or vascular diseases can also cause hemoptysis [1]. We present an unusual case of hemoptysis as a delayed complication of scoliosis surgery. This work has been reported in line with the SCARE criteria [2]. Informed consent was obtained from the patient for the publication of this case report.

## 2 Presentation of case

**Fig. 1:**
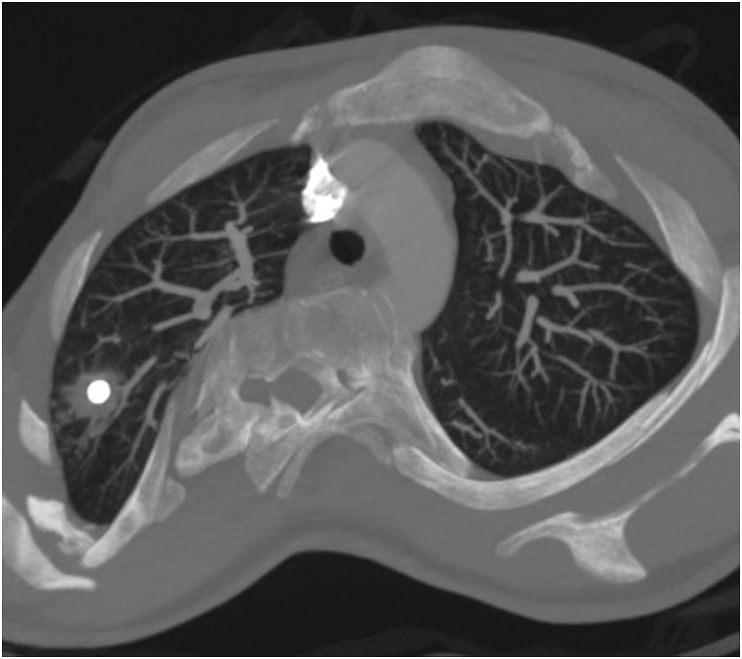
Axial maximum intensity projection (MIP) CT image shows the upper edge of spinal rod within the right upper lobe surrounded by consolidations corresponding to injured parenchyma.

**Fig. 2:**
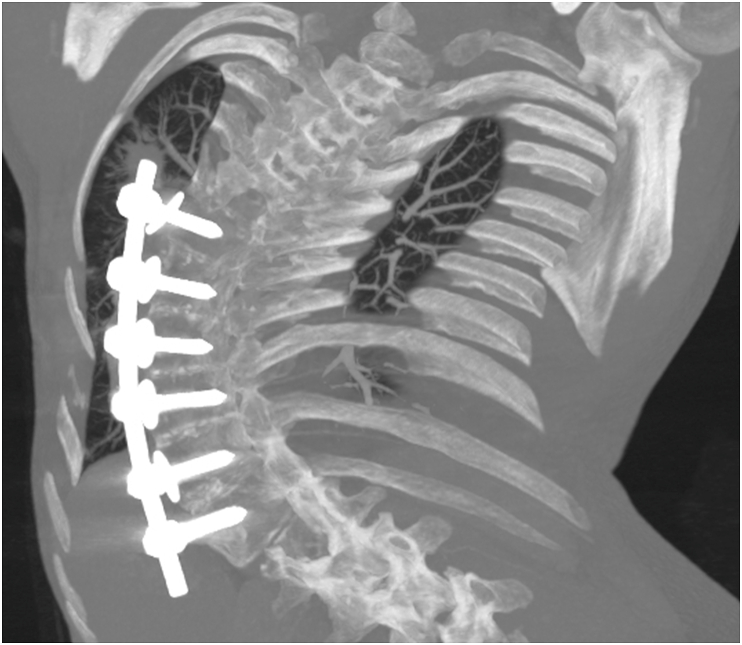
Oblique coronal MIP CT image shows displaced anterolateral spinal rod and adjacent screw to the right upper lobe; the caudal edge of the rod penetrated to the liver.

**Fig. 3:**
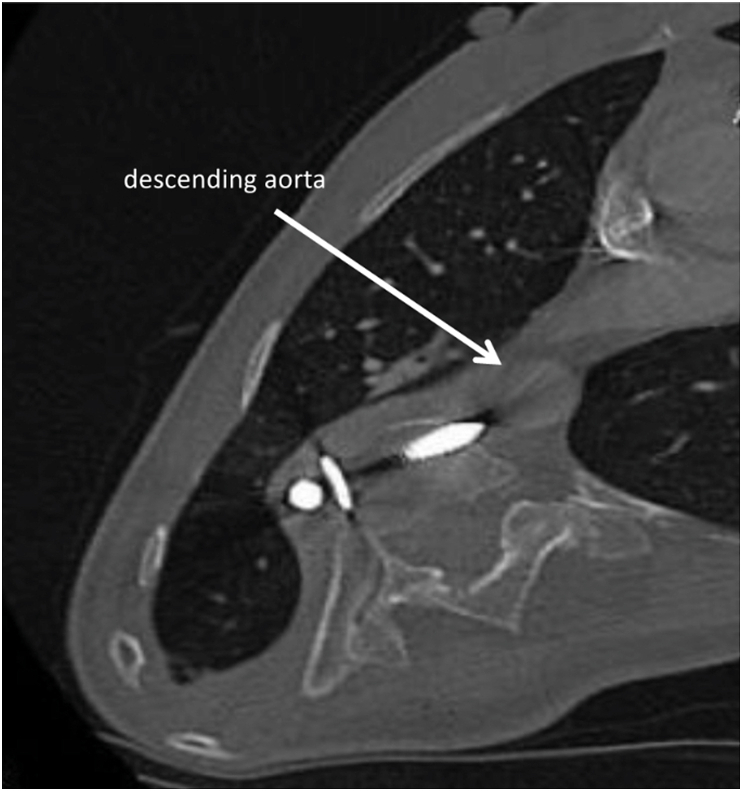
Axial CT image (bone window) shows the tip of the screw penetrating to the posterior mediastinum, close to descending aorta.

A 30-year-old woman was admitted to our institution in 2019 for hemoptysis. She had a history of scoliosis and had undergone surgical antero-lateral Th7 through Th12 spinal fusion 18 years earlier. She was a smoker, but denied any history of drug abuse or the inhalation of intoxicants. In the past, she had 2 pregnancies - in 2010 and 2018 - terminated by caesarean section. She had three episodes of hemoptysis during the year. The patient was expectorating sputum mixed with a small amount of fresh blood the size of a few pin heads, without chest pain or dyspnea. Hemoptysis resolved spontaneously or after empirical antibiotic therapy, and the patient did not require emergency treatment or admission. Few days before hospitalization at our institute, the hemoptysis was more pronounced – she expectorated about 5–10 mL of blood daily, and complained of chest tightness. At admission, the patient's general condition was good. Physical examination revealed advanced scoliosis of the thoracic and lumbar spine to the right, blood pressure 110/60 mmHg, pulse 82 bpm, respiratory rate 14/min, saturation 97%, and body temperature 36.5 C. Results of laboratory tests showed no abnormalities, C-reactive protein (CRP) was normal, there was no anemia, and the clotting parameters (prothrombin time, PT; activated partial thromboplastin time, APTT) were normal. Chest computed tomography (CT) showed penetration of the spinal rod into the right upper lobe of the lung; around the edge of the rod pulmonary consolidations and nodules corresponding to lung injury were visible (Fig. 1). The caudal edge of the rod penetrated into the liver (Fig. 2), while the joining screws crossed the cortex of the vertebral bodies and moved into the posterior mediastinum close to the descending aorta (Fig. 3). Hemoptysis was the result of penetration of the rod into the lung. Due to the high risk of complications, bronchoscopy was abandoned. The patient was urgently referred to the Orthopedics Department and underwent extensive surgery to remove the spinal rod. The surgery required the cooperation of an orthopedist, thoracic and general surgeon, and was completed successfully. During the 6 months of follow-up, the patient's clinical condition was good, with no recurrence of hemoptysis, and no deterioration in mobility. The patient remains under the care of orthopedists, who are considering a second intervention for stabilization of the spine.

## 3 Discussion

Scoliosis is defined as a lateral curvature of the spine of ≥10ͦ in the coronal plane. Surgery is recommended when the curvature reaches 45ͦ to 50ͦ or if curves progresses at an accelerated rate. The goals is to correct the deformity, prevent progression of the curve, restore trunk symmetry, and minimize pain and morbidity [3]. After successful surgery, many adolescents can return to diverse activities [4].

Although modern instrumentation and new surgical techniques result in good surgical outcomes, the complication rates have remained somewhat constans ranging between 5% and 23% [5,6]. Delayed complications identified on radiogtaphy most commonly include broken rods and pseudarthrosis requiring re-instrumentation [7]. Incorrect placement of the pedicle screw has been reported in 15.8% of the patients, while intrathoracic bleeding is rare and usually occurs intraoperatively [8].

Delayed migration of the scoliosis rod is an exceedingly rare complication. However, in most cases, passage of the rod into the rectum or the lower extremity [9,10], as well as into the iliac fossa [11] or spinal cord resulting in compression and neurological injury have been reported [12,13]. Penetration of the rod into the lung – as observed in our patient - is an extremely rare condition. There are only few similar reports in the literature. Song et al. [14] reported transdiaphragmatic migration of the rod into the left lung 14 years after antero-lateral spinal fusion. Wong et al. [15] described the case of a 25-year-old man with hemoptysis due to penetration of a Dwyer cable into his right bronchus 9 years after insertion for idiopathic scoliosis. Interestingly, in that case hemoptysis persisted for 4 years, before the diagnosis was established. The patient had been previously investigated, but the condition was diagnosed as bronchitis. In our case, hemoptysis recurred for a year, before the patient was sent for investigations, and similar to that in the patient mentioned above, it was attributed to respiratory tract infection. Chest CT scan, performed on the first day of hospitalization, identified the cause of hemoptysis. The scan revealed penetration of the rod into the lung, while its caudal edge penetrated into the liver, and the joining screws moved to the mediastinum close to the descending aorta. To our knowledge, there are no other reports of multiorgan passage of the spinal rod.

The complexity of spinal surgery is reflected in the diversity of complications that maight occur months or years later [8,16]. Spinal rod migration happens rarely, and among the reasons mentioned are repetitive fatigue or minor trauma [9], fatigue-fracture of the rod secondary to motion in the setting of nonunion of fusion attempts [14], or progressive kyphosis [15]. In our patient symptoms appeared 18 years after surgery and were attributed to respiratory tract infection. Perhaps, the pregnancies had an adverse effect on the implanted spine stabilizing system. Indeed, some authors reported an increased risk of progression of scoliosis associated with pregnancy in patients operated using Harrington instrumentation [17]. However, some recent reports of women who have underwent pedicle screw instrumentation and fusion showed that pregnancies did not lead to curve progression in the long-term [18]. There is no specific data or explanation of the pathophysiology of deterioration in spine stabilization during pregnancy. The physical burden that a fetus places on the spine of the mother with underlying scoliosis might lead to curve progression and – as a consequence - rod migration [17]. Li et al. [19] described a case of revision surgery after pregnancy (4 months after the patient gave birth) in a patient with congenital kyphoscoliosis, in who the spinal rod was broken. The authors reported that the weight gain of pregnancy was the most likely reason for the rod breakage. Diagnosis of the complications of scoliosis surgery is additionally more difficult because the early stages of rod migration are often asymptomatic [9], therefore, we recommend systematic follow-up with clinical and radiological examination to prevent or minimize such complications.

Treatment of hemoptysis is challenging for physicians, because in about 20% of the cases and in up to 42% of the smokers, an etiological diagnosis cannot be established even after bronchoscopy and chest CT [20]. It has multiple causes, but spinal rod penetration into the lung, is extremely rare. Usually, an assessment of the clinical significance of hemoptysis should consider the volume of expectorated blood and its effects on the respiratory and cardiovascular reserves of the patient [1]. Our patient presented with slight hemoptysis and had no other complaints. Furthermore, her general condition was good and the episodes of hemoptysis temporarily resolved; this might have led to reduced vigilance by the physicians’. She was referred for investigations only one year after the persistence of hemoptysis. Our case indicated that every incidence of hemoptysis should be promptly investigated, because it can be life-threatening. Any further delay in the diagnosis in our patient could have resulted in massive bleeding and death.

## 4 Conclusion

In conclusion, this case highlights several important issues: 1) it demonstrates an unusual cause of hemoptysis due to penetration of the rod inserted during surgery for scoliosis into the lung 18 years after surgery; this condition was accompanied by migration the caudal edge of the rod into the liver and displacement of the joining screws into the mediastinum threatening to damage the aorta, 2) it highlights the need to promptly and thoroughly evaluate patients with even slight hemoptysis because it can be life-threatening, 3) it indicates the need for periodic follow-up of patients after scoliosis surgery to prevent or minimize its complications, and, finally 4) this unusual case describes successful treatment despite the rare and potentially morbid complication.

## Provenance and peer review

Not commissioned, externally peer reviewed.

## Ethical reports

Ethical approval has been exempted by our institution for reporting this case.

## Sources of funding

No founding was sought or secured in relation to this case report.

## Consent

Written informed consent was obtained from the patient for the publication of this case report and accompanying images. A copy of written consent is available for review by the Editor-in-Chief of this journal on request.

## Author contribution

All authors have contributed to conception and design of the study, drafting the article, revising it critically for important intellectual content. All authors have approved the final article.

## Trials register

Not applicable.

## Guarantor

Justyna Fijolek.

## Declaration of competing interest

The authors declare that they have no conflict of interest regarding the publication of this case report.
